# Dostarlimab: A Review

**DOI:** 10.3390/biom12081031

**Published:** 2022-07-26

**Authors:** Bárbara Costa, Nuno Vale

**Affiliations:** 1 OncoPharma Research Group, Center for Health Technology and Services Research (CINTESIS), Rua Doutor Plácido da Costa, 4200-450 Porto, Portugal; bcosta@med.up.pt; 2 CINTESIS@RISE, Faculty of Medicine, University of Porto, Alameda Professor Hernâni Monteiro, 4200-319 Porto, Portugal; 3 Department of Community Medicine, Information and Health Decision Sciences (MEDCIDS), Faculty of Medicine, University of Porto, Rua Doutor Plácido da Costa, 4200-450 Porto, Portugal

**Keywords:** anti-PD-1 antibody, dostarlimab, immunotherapy, clinical trials

## Abstract

Dostarlimab (JEMPERLI) is a PD-1 monoclonal antibody for the treatment of adult patients, with mismatch repair deficient (dMMR), recurrent or advanced endometrial cancer that has progressed on or following prior therapy with a platinum-containing regimen. As determined by an FDA-approved test this indication was granted rapid approval based on the rate of tumor response and the duration of the response. Continued approval for this indication is conditioned on further confirmatory trials demonstrating and documenting clinical benefit. In June 2022, the clinical trial NCT04165772 reported a 100% remission rate for rectal cancer. This clinical trial brought proof that we can match a tumor and the genetics of what is driving it, with therapy. This clinical trial continues to enroll patient and is currently enrolling patients with gastric, prostate, and pancreatic cancers. Dostarlamib is being recommended for rectal cancer. The focus of this review is to summarize the existing knowledge regarding Dostarlimab and explore the possibilities of mono- and combination therapies.

## 1. Introduction

In 1986, the first immunotherapy agent, an antitumor cytokine designated interferon-alpha 2 was approved by the US Food and Drug Administration (FDA). IFN-a2 was first approved for the treatment of hairy cell leukemia (HCL) after studies showed that it had a high response rate in patients with advanced HCL. In 1995, the FDA approved IFN-a2 for use as adjuvant therapy for stage IIB/III melanoma. When it was licensed for the treatment of metastatic melanoma and renal cell carcinoma in 1998, interleukin-2 (IL-2), a T-cell growth factor that aids in immunological modulation and T-cell proliferation, became the second anticancer cytokine approved by the FDA. Since the development of immunotherapies a promise of revolutionizing the standard care in cancer treatment has existed and, in recent years, a novel class of immunotherapeutics known as checkpoint inhibitors has emerged as a cornerstone in cancer treatment [1]. To this day, different types of immunotherapies are used to treat cancer: immune checkpoint inhibitors, T-cell transfer therapy, monoclonal antibodies, vaccines, and immune system modulators.

Notably, a record number of antibody therapeutics have been granted approval in either the European Union (EU) or the United States (US). In diseases such as cancer, immunotherapies have drastically changed the game for patients, since immunotherapies get the immune system properly engaged to eradicate cancer cells. For example, the use of programmed cell death protein-1 (PD-1) and the cytotoxic T-lymphocyte-associated protein-4 (CTLA-4) has been demonstrating increased median overall survival and durable responses in patients across multiple tumors. Thousands of people have benefited from immune checkpoint inhibitors (ICPIs); however, despite long-lasting responses in a variety of tumor types, most patients either do not respond at all or develop resistance to the ICPI. Additionally, ICPI treatment has the potential to cause major side effects, and therefore identification of patient populations that will benefit from ICPI as single medicines and in combination is urgently needed [2].

Regarding activated T cells, PD-1 is an inhibitory immunological checkpoint receptor. PD-1 reduces activated effector T-cell capabilities such as proliferation, cytokine generation, and cytotoxic activity by interacting with its ligands, programmed cell death ligands 1 and 2 (PD-L1 and PD-L2). One of the strategies through which tumor cells elude the immune system and interfere with cancer-specific immune responses is the upregulation of PD-L1. Preclinical and clinical investigations have shown that treatments that bind to either the PD-1 receptor or ligand and effectively disrupt the receptor–ligand interaction can boost antitumor immunity and improve patient survival in a range of malignancies [3]. So far, the FDA has approved six PD-1 and PD-L1 inhibitors for clinical usage, collectively known as PD-L1. Patients with cancer can choose from a variety of dose regimens, disease-specific treatments, tolerance profiles, and pricing alternatives due to the competitive environment of anti-PD-1 antibodies.

**Figure 1:**
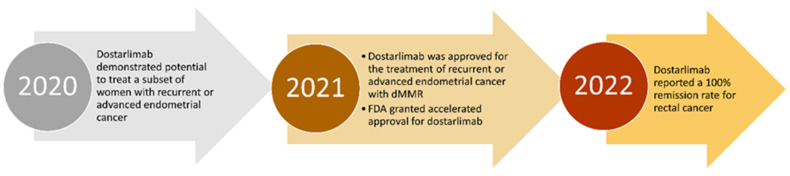
Timeline of the drug Dostarlimab since the demonstrated potential to treat a subset of women with EC to the reported 100% remission rate in rectal cancer.

On 17 August 2021, the FDA granted accelerated approval to Dostarlimab, a monoclonal antibody, for adults with dMMR recurrent or advanced endometrial cancer that has progressed despite ongoing or prior treatment with the platinum-containing chemotherapy regimen, Figure 1. Tumors that exhibit the dMMR or MSI-H biomarker have an abnormal function of DNA repair mechanisms. Genes that should repair any improper activity to maintain cell health are absent in these types of cancer. Dostarlimab, an inhibitor of PD-1, demonstrated a long-lasting effect on dMMR tumors, and in 2022, reported a 100% remission rate for rectal cancer [4]. All patients had dMMR, a mutation present in 5 and 10% of rectal cancer cases (this mutation is also present in endometrial, prostate, and bladder tumors). This clinical trial brought the promise and the proof that we can match a tumor and the genetics of what is driving it, with therapy.

### 1.1. About the Drug

Dostarlimab (also referred to as TSR-042 or Jemperli, commercial name) is a humanized mAb of the IgG4 isotype, produced by recombinant DNA technology in mammalian Chinese hamster ovary (CHO) cells that binds PD-1 on T cells and blocks interactions with its ligands PD-L1 and PD-L2, activating immune responses. Dostarlimab is an immunotherapy that aids the body’s natural anti-tumor immune response during cancer treatment. It is given via intravenous infusion for over 30 min every three to six weeks, depending on the cycle.

To prevent the formation of half-antibodies, each heavy chain of the antibody has a serine to proline substitution (S228P) to promote the stabilization of disulfide bonds between the two heavy chains. Dostarlimab was humanized by grafting the heavy- and light-chain complementarity-determining regions on the germline variable region frameworks of their nearest CONTACT human species orthologs, followed by affinity maturation via mammalian cell display and somatic hypermutation, using the AnaptysBio SHM-XEL system. The company Anaptysbio developed the drug Dostarlimab, also in collaboration with Tesaro, and was bought by GlaxoSmithKline in 2019 [5,6]. The Jemperli^TM^ final product is a concentrate for infusion solution containing 500 mg of dostarlimab as the active ingredient. Trisodium citrate dihydrate, citric acid monohydrate, L-arginine hydrochloride, sodium chloride, polysorbate 80, and water for injection are among the other constituents. 

Although the heavy chain of Dostarlimab is involved in the interaction between PD-1 and Dostarlimab, the light chain is predominantly responsible for steric blockage of PD-L1 binding. To attain high affinity, Dostarlimab causes conformational rearrangements in the BC, C’D, and FG loops of PD-1. By occupying the concave surface on the heavy chain via numerous interactions, the residue R86 within the C’D loop of PD-1 plays a vital role in Dostarlimab binding. This high-resolution structure could be useful in developing better anti-PD-1 biologics or effective cancer immunotherapy combination methods [7]. Dostarlimab has a KD value of 0.3 nM for human PD-1, with an association rate of 5.7105 (M^−1^s^−1^) and a dissociation rate of 1.7 104 (s^−1^), indicating fast target association and delayed dissociation [5]. Since IgG4 isotypes elicit modest Fc-mediated effector functions such as antibody-dependent cellular cytotoxicity (ADCC) and complement-dependent cytotoxicity (CDC), Dostarlimab was designed to avoid tumor-reactive T cell depletion. Fc binding has been used to illustrate the lack of Dostarlimab ADCC activity (in a Biacore analysis).

The structural basis is still unrevealed, but it is known that an epitope within a target molecule might be a critical component of a therapeutic antibody since antibodies that recognize different epitopes have varying therapeutic efficacy. Even though that antibodies against PD-1 and PD-L1 have a similar blocking function, they identify different antigenic epitopes. Because of their high specificity and affinity for their targets, monoclonal antibodies have been a key therapeutic method for decades. The high-resolution structure revealed that Dostarlimab binds to the flexible loops of PD-1, including the BC, C’D, and FG loops, differently than Pembrolizumab or Nivolumab [7].

Moreover, Dostarlimab was characterized by a variety of in vitro and in vivo experiments, as well as preclinical actions that enabled it to become an investigational new drug. Dostarlimab has no cross-reactivity with the mouse orthologue, and it does not cause considerable cytokine stimulation when used alone [5]. It was first studied in single-dose tests, followed by 4-week repeat-dose study, and subsequently a 13-week repeat-dose study. All the evidence pointed to Dostarlimab being well tolerated at doses of 30 and 100 mg/kg, with toxicity comparable to other anti-PD-1 antibodies [8]. Dostarlimab showed anticancer effectiveness, as measured by tumor growth inhibition, which was linked to enhanced immune cell infiltration. These findings show that Dostarlimab is a strong anti-PD-1 receptor antagonist with features that warrant further clinical testing in cancer patients.

Dostarlimab has an anti-drug antibodies (ADA) rate of 2.5%, which, again, is comparable to other anti-PD-L1 medicines, and it only induces a modest immune response in a limited fraction of cancer patients after one or more treatment cycles. Dostarlimab’s high product purity and mode of administration reduces the danger of inducing immunological reactions. Furthermore, there is currently no evidence that pre-existing ADAs or the generation of ADAs has any effect on any safety or efficacy measurements. These findings suggest that Dostarlimab is a novel and effective anti-PD-1 monoclonal antibody with a low risk of eliciting immunogenic reactions [9]. Data from the GARNET trial support reports that dMMR/MSI-H is a predictive biomarker of response to anti-PD-L1 agents; however, Dostarlimab has shown clinical activity in endometrial cancer (EC) and nonsmall cell lung cancer (NSCLC) regardless of MMR status, with a tolerability profile similar to other anti-PD-1 mAbs across tumor types.

### 1.2. Effect on T-Cell Activation: Preclinical and Clinical Characterization

Dostarlimab was found to improve T-cell activation in a variety of in vitro functional test methods using primary human T cells. While Dostarlimab improved T-cell activation in antigen-dependent systems, it had no direct (nonspecific) effects on T-cell responses, as seen by the lack of cytokine production in the absence of antigen. Dostarlimab was found to have effective anticancer action in humanized mice tumor models, as well as a consistent pharmacokinetic and pharmacodynamic profile with negligible off-target effects. Its anticancer effectiveness was linked to a decrease in tumor-associated regulatory T cells and an increase in tumor-infiltrating CD8+ T cells [10]. For example, dostarlimab treatment in vitro decreased tumor growth in the MDA-MB-436 breast cancer model (TGI of 53%) when compared to isotype control [5].

Dostarlimab’s anti-PD-1 antibody profile was shown to be good in preclinical testing, with effective binding to PD-1 and antagonizing interactions with PD-L1 and PD-L2. Dostarlimab binds to the human PD-1 receptor with a high affinity, with a binding affinity (KD) of 300 pM. Preclinical findings for the previous approved PD-1 treatments such as Nivolumab, Pembrolizumab, and Cemiplimab have a similar binding profile. Dostarlimab was chosen for its IgG4 isotype to generate the most dependable and efficacious therapy. Other anti-PD-1 antibodies (Pembrolizumab, Nivolumab, and Cemiplimab) are all IgG4 modalities, but anti-PD-L1 antibodies (Atezolizumab, Avelumab, and Durvalumab) are all IgG1 modalities (added risk is a possibility in the chronic administration of IgG1 Fc for immunological enhancement). According to surface plasmon resonance, flow cytometry employing cell lines overexpressing recombinant PD-1, or binding to the native protein on peripheral blood mononuclear cells, Dostarlimab bound to both human and cynomolgus monkey PD-1 with great affinity [11]. The antibody also prevented PD-L1 and PD-L2 from interacting with the receptor. In a human CD4+ mixed lymphocyte response assay, Dostarlimab, acted as a powerful functional antagonist, resulting in enhanced IL-2 production. In this test, the addition of anti-TIM3 or anti-LAG3 antibodies increased the activity of Dostarlimab. Dostarlimab incubation of human peripheral blood mononuclear cells (PBMCs) as a single agent did not result in significant cytokine release stimulation.

Interfering with the PD-1/PD-L1 pathway removes an essential immune system inhibitory response, which can lead to severe or fatal immune-mediated adverse effects. These reactions can occur in any organ system and at any time after starting therapy; while they are most common during therapy, they can also occur after the causative substance is stopped. Patients on Dostarlimab should be closely monitored for signs of an underlying immune-mediated reaction, and if one is suspected, they should be assessed and treated immediately. It is important to remember that anti-PD-L1 therapies are associated with a multitude of side effects, including pneumonitis, hypothyroidism, colitis, and infusion-site responses. Although enhancing the immune system’s activation state is an effective anticancer strategy these events are most likely connected to the target’s binding and associated pharmacodynamic consequences, such as anti-PD-L1 treatments’ immune-related side events. The cytotoxicity caused by the antibody’s binding to complement, or FC receptors is another important factor in predicting the safety profile of a new anti-PD-(L)1 treatment. Dostarlimab demonstrated little to no binding to Fc or a complement protein C1q, receptors that induce ADCC, and CDC, respectively, and so is unlikely to result in the depletion of antitumor effector T cells, which is consistent with its IgG4 framework.

The preclinical data supported Dostarlimab first-in-human dose selection and showed that the drug had a sufficient safety margin to be examined further in the Phase 1 GARNET trial’s human dose-finding sections 1 and 2A. Dostarlimab has shown significant and sustained responses in ongoing clinical studies, as well as a manageable safety profile with side effects like those seen with other anti-PD-1 treatments. No dose-limiting toxicity was observed. Dostarlimab has shown potential as an anti-PD-1 therapy in different clinical studies, including RUBY (NCT03981796), FIRST (NCT03602859), IOLite (NCT03307785), and MOONSTONE (NCT03307785), and several more, where it is being tested either as a monotherapy and in combination for a variety of tumor types (NCT03955471) [12].

### 1.3. Pharmacodynamics and Pharmacokinetics 

A population PK (PopPk) profile of Dostarlimab was well described by a 2-compartment model with time-dependent linear elimination. At clinically relevant doses, the PopPK model revealed that Dostarlimab exposure is approximately dose-proportional. The PK profile of Dostarlimab is generally consistent with that of other approved PD-1 inhibitors, Pembrolizumab, Nivolumab, and Cemiplimab because PK parameters were similar, and both time-varying CL and a linear elimination pattern were previously observed for these agents within their therapeutic dose range. While Dostarlimab’s time-varying CL is similar to other PD-1 inhibitor observations, the typical maximum drop in CL over time was calculated at 14.9%, which is lower than that reported for Pembrolizumab (20–30%), Nivolumab (25%), or Cemiplimab (35.9%). Interestingly, in stepwise covariate modeling, tumor type (EC (dMMR/MSI-H), NSCLC or MSI-H, etc.) was not determined to be a statistically significant covariate and did not affect Dostarlimab PK characteristics. Body weight and time-varying albumin were observed to influence Dostarlimab PK, as previously reported for other PD-1 inhibitors. The impact of body weight on exposure was considered not clinically relevant. For short, patient covariates/disease characteristics had limited clinically relevant effects on exposure.

Dostarlimab has a pharmacokinetics (PK) profile that permits the dosing interval to be increased from three to six weeks. The pharmacodynamic activity of Dostarlimab was conducted in both in vitro and in vivo experimental systems. Throughout the first cycle 500 mg was administered intravenously every 3 weeks, and the absorption was described by: the mean Cmax and AUC0-tau of dostarlimab as 171 mcg/mL and 35,730 mcg.h/mL, respectively. When administered at 1000 mg every 6 weeks, the mean Cmax and AUC0-tau are 309 mcg/mL and 95,820 mcg.h/mL, respectively. At steady state, the mean volume of distribution of Dostarlimab is 5.3 L. The metabolism of Dostarlimab has yet to be characterized, but for now it is estimated to be degraded via catabolic pathways into smaller peptides and amino acids [13]. The mean terminal elimination half-life of Dostarlimab is 25.4 days, and the mean clearance of Dostarlimab is 0.007 L/h. There are no data regarding overdose with Dostarlimab. Symptoms of overdosage are likely to be consistent with the adverse effect profile of Dostarlimab and may therefore involve significant immune-mediated reactions [14,15].

Other authorized PD-1 inhibitors have the following PK characteristics: long half-life, limited extravascular diffusion, and minimal impact of hepatic or renal function impairment on PK [16]. The anti-PD-1 class is also characterized by time-varying drug clearance (CL). However, there are some discrepancies in the PK properties of anti-PD-1 drugs that may reflect target-mediated drug disposition. PD-1 inhibitors Pembrolizumab and Nivolumab have fixed and body weight dosage regimens, depending on their permitted indications; PD-1 inhibitor Cemiplimab has a fixed dosing schedule. Using data from the GARNET trial the PK and exposure-response (ER) studies of Dostarlimab in patients with recurrent/advanced solid tumors were described. One of the goals was to identify clinically important variables and assess ER correlations for overall response rate (ORR) and the occurrence of relevant adverse events (AEs) [15,17]. 

Noticeable, there is a correlation between tumor mutational burden status (TMBST) and dMMR/MSI status in some tumor types, including EC and colorectal cancer, and multivariate logistic regression analysis revealed that the impact of TMBST on overall response rate (ORR) was significant for the entire dataset and the EC subgroup. A similar correlation between TMBST and ORR has previously been reported for immune-checkpoint inhibitors [15]. There are no known drug–drug interactions with Dostarlimab, as it is a monoclonal antibody and thus is not a cytochrome P450 or drug transporter substrate, and is unlikely to be a cytokine modulator [18].

## 2. Immune Microenvironment and Emerging Treatments 

T cells, B cells, natural killer cells, and other tumor-infiltrating lymphocytes express PD-1, a transmembrane receptor. Antigen-presenting cells (APCs) and certain nonimmune cells, particularly tumor cells, express PD-L1 and PD-L2. The immunological inhibitory checkpoint PD-1 and its ligands are involved in T-cell activation and tolerance [19]. The binding of PD-L1 or PD-L2 to PD-1 prevents lymphocyte activation and improves immunological tolerance to self-antigens to prevent tissue injury, but it also prevents immune cells from responding to tumors. Tumors have been demonstrated to use the PD-1 signaling pathway to elude immune regulation and enhance tumor growth by upregulating PD-L1 expression. Even though mAbs have proven to be highly effective immuno-oncology therapy agents in clinical trials, there are still obstacles to their general usage in all patients, such as the rise of immunogenicity. ADAs often have no overall clinically relevant effects, although they can affect pharmacokinetics and have an impact on safety or efficacy. Patient-related factors (e.g., human leukocyte antigen type, disease, concomitant drugs, and immunological competence), dosing regimen, method of administration, and essential product factors all have a role in the development of ADAs [20]. Moreover, infusion reactions, hypersensitivity reactions, anaphylaxis, and ADA-mediated diseases are all examples of how ADAs can reduce efficacy by altering drug clearance. They can also have an impact on drug safety through infusion reactions, hypersensitivity reactions, anaphylaxis, and ADA-mediated diseases. Neutralizing antibodies (NAbs), another form of ADA, diminish efficacy by disrupting target binding. Even though the humanization of antibodies reduces the risk of antidrug immunological reactions, immunogenic responses are nevertheless seen in both partially and fully humanized antibodies [21].

Pembrolizumab, Nivolumab, Cemiplimab, Atezolizumab, Avelumab, and Durvalumab are mAbs that disrupt the interaction of PD-1/PD-L1 and thereby eliminate cancers’ ability to evade the immune system [9]. Nivolumab (OPDIVO^®^) and Pembrolizumab (KEYTRUDA^®^) were approved by the US FDA in 2014 for the treatment of melanoma and nonsmall cell lung cancer. Since then, the antibodies’ indications have been expanded to include renal cell carcinoma, classical Hodgkin lymphoma, squamous cell carcinoma of the head and neck, urothelial carcinoma, esophageal carcinoma, endometrial cancer, squamous cell carcinoma, hepatocellular carcinoma, and breast cancer, either as monotherapy or in combination with other drugs, Table 1. These drugs are also indicated for dMMR/MSI-H testing. Dostarlimab, the fourth PD-1 monoclonal antibody, was approved in 2021 [7]. Scientific advancements have facilitated the development of mAb therapeutics that entered clinical trials and were granted marketing approvals.

In 2019, GSK anticipated potential regulatory submissions for Dostarlimab, after the results of Phase I dose-escalation and cohort expansion study (GARNET; NCT02715284). This study evaluated the safety and efficacy of Dostarlimab monotherapy for patients with advanced solid tumors, including women with recurrent or advanced endometrial cancer who progressed on or after a platinum-based regimen. Patients were administered 500 mg every 3 weeks for the first 4 cycles, and 1000 mg every 6 weeks. Of the 25 patients with microsatellite instability in endometrial cancer, one had a complete response and 12 had partial responses. The objective response rate was 43.5% in this group of 108 patients, according to RECIST v1.1 (47/108; 95% confidence interval: 34.0–53.4), with 10.2% complete responses (CRs) (11/108) and 33% partial responses (36/108). Furthermore, 89.4% of respondents had an active answer at the time of data cut-off [22].

Early-stage clinical studies, as well as two Phase 3 studies, RUBY and FIRST, are evaluating Dostarlimab as a treatment for several forms of cancer. The Phase 3 RUBY study (NCT03981796) is a 2-part study. Part 1 is to evaluate the efficacy and safety of Dostarlimab plus carboplatin-paclitaxel followed by Dostarlimab versus placebo plus Carboplatin-Paclitaxel followed by placebo. Part 2 is to evaluate the efficacy and safety of Dostarlimab plus Carboplatin-Paclitaxel followed by Dostarlimab plus Niraparib versus placebo plus Carboplatin-Paclitaxel followed by placebo in participants with recurrent or primary advanced (Stage III or IV) endometrial cancer. TSR-042 is also being tested in a Phase 3 FIRST trial (NCT03602859), which compares platinum-based therapy with TSR-042 and Niraparib to the standard of care platinum-based therapy as first-line treatment of Stage III or IV non-mucinous epithelial ovarian cancer [8].

The EMA approved the conditional marketing authorization since Jemperli answers an unmet medical need and the benefit of immediate availability outweighed the risk of less comprehensive data than is generally necessary. In 2021, either the US or the EU granted accelerated approvals to 11 antibody therapeutics. The FDA granted accelerated approval to five of the seven products, Dostarlimab, Loncastuximab Tesirine, Amivantamab, Aducanumab, and Tisotumab vedotin. Jemperli received an additional accelerated approval from the FDA on 17 August 2021, for the treatment of adult patients with dMMR recurrent or advanced solid tumors, as determined by an FDA-approved test, that have progressed on or following prior treatment and who have no satisfactory alternative treatment options. This approval was based on the tumor response rate and the response’s duration. The increased number and variety of antibody therapies that may be approved soon will almost certainly have a significant impact on patient care. This is particularly true for cancer patients, who may soon have access to a significantly higher number of antibody immune checkpoint modulators and antibody–drug conjugates.

### 2.1. Inhibitors of PD-1/PD-L1 and dMMR

Cancer immuno-therapy has seen significant clinical success driven by ICBs that restore T-cell activation. ICBs act in multiple ways to alter T-cell function, including the downregulation of inhibitory signaling [14]. One target of ICBs is programmed cell death protein 1 (PD-1). Multiple malignancies have high levels of PD-L1 and PD-L2, which suppress T cells. Monoclonal antibodies that target PD-1 or PD-L1 (PD-(L)1) disrupt the interaction between PD-1 on T cells and PD-L1 on cancer cells, restoring T-cell activity. The PD-L1 inhibitor has been licensed as an immunotherapeutic for a variety of malignancies [23], see Table 1.

Anti–PD-(L)1 pathway-targeted treatments have been demonstrated to be well tolerated and have consistent safety profiles as a pharmacological class, and when used to treat dMMR-MSI-H, PD-1/PD-L1 inhibitors showed favorable clinical results, including a higher response rate. However, not all drugs entitled of PD-1/PD-L1 inhibitors have the same success rate in treating tumors with dMMR. In Sclafani’s review, it is highlighted that the administration of Pembrolizumab to patients with dMMR metastatic colorectal cancer was correlated with a poor prognosis. Numerous studies have demonstrated the wide range of immunotherapy, prognosis, and chemotherapy sensitivity in individuals with dMMR/MSI malignancies, and the detection limitation contributes to the difficulty of treatment. Moreover, it is necessary to quantify the frequency of missmatch repairs. A lower or higher frequency will require a different treatment. However, it remains unclear how the same PD-1/PD-L1 inhibitors (or even different ones) cause variable therapeutic responses in patients with a different frequency of mismatch repairs [24]. At last, it appears Dostarlimab that shows durable antitumor activity in patients with dMMR/MSI-H.

### 2.2. Combination Studies

Based on the enormous success of antibodies targeting PD-1 or its ligand PD-L1, the low response rate of -PD-1/PD-L1 therapy must be addressed. For most cancer patients, the PD-1/PD-L1 pathway is not the only mechanism limiting antitumor immunity and inhibiting the PD-1/PD-L1 axis is insufficient to generate an effective antitumor immune response. Some combination therapies, such as PD-1/PD-L1 plus chemotherapy, radiation, angiogenesis inhibitors, targeted therapy, additional immune checkpoint inhibitors, co-stimulatory molecule agonists, interferon gene stimulator agonists, fecal microbiota transplantation, epigenetic modulators, or metabolic modulators exhibit better response rates and superior anticancer efficacies [12], see Table 2.

For example, Belamaf (Belantamab mafodotin) is a B-cell maturation antigen (BCMA)-targeted antibody–drug combo that was recently licensed as monotherapy for people with relapsed/refractory multiple myeloma. For patients with relapsed/refractory multiple myeloma, a phase I/II platform research comparing the safety and efficacy of belamaf combination with Dostarlimab (a PD-1 blocker) to belamaf monotherapy is underway [25]. BCMA is a target found on tumor cells in multiple myeloma patients. Belantamab mafodotin is an ADC that contains a humanized anti-BCMA monoclonal antibody (mAb). 

Combination techniques have been developed to generate synergistic effects or to diminish primary or secondary resistance to PD-L1 inhibitors due to the complexity of immune response activation and the multiple mechanisms leading to resistance to PD-(L)1 inhibitors. Combinations with CTLA-4, TIGIT, IDO, and PVRIG are being evaluated in early clinical trials to block other immune checkpoints (NCT03015129, NCT04570839, NCT04106414, NCT03667716) [25], and future findings will give insight into their therapeutic utility in this environment. In the recurrent scenario, angiogenesis and PARP inhibitors are investigated, whereas chemotherapy is investigated in the first-line setting.

Pembrolizumab and Dostarlimab have shown impressive results in MMR-deficient cases, and the association of Pembrolizumab and Lenvatinib is becoming a standard of care for pretreated recurrent MMR-proficient EC. However, further advances are needed to understand primary and secondary mechanisms of resistance to immunotherapy and to implement ICI in the first-line metastatic setting and early-stage tumors.

Patients with platinum-resistant ovarian cancer have a poor prognosis and few therapy alternatives. In this group of patients, preclinical and clinical studies showed that combining poly-ADP ribose polymerase inhibitors with immune checkpoint drugs could have a synergistic anticancer effect (NCT04679064) [26]. Moreover, the phase IB trial evaluates the effect of Niraparib and Dostarlimab in treating patients with BRCA-mutated breast, pancreas, ovary, fallopian tube, or primary peritoneal cancer that cannot be removed by surgery (unresectable) or has spread to other places in the body (metastatic). Niraparib is an inhibitor of PARP, an enzyme that helps repair deoxyribonucleic acid (DNA) when it becomes damaged. Blocking PARP may help keep cancer cells from repairing their damaged DNA, causing them to die. PARP inhibitors are a type of targeted therapy. Immunotherapy with monoclonal antibodies, such as TSR-042, may help the body’s immune system attack cancer and may interfere with the ability of tumor cells to grow and spread. Giving Niraparib and TSR-042 may kill more cancer cells (NCT04673448).

For advanced solid tumors, IOLite is a dose-finding trial of Dostarlimab in combination with the PARP inhibitor Niraparib or platinum-based chemotherapy Bevacizumab. It has four arms, each of which is adorned with Dostarlimab. Patients were assigned to either arm based on the histology of their tumors, their prior treatment history, and their physician’s recommendation. In diverse forms of cancer (ovarian, small cell lung cancer, breast, bladder, prostate, endometrial, and NSCLC), there was one complete response with the combination of Dostarlimab plus chemotherapy, and there were partial responses in any of the four arms. There were no pharmacokinetic interactions between Dostarlimab and Niraparib and the combination. As a result, the combination of the two medications appears to be successful, with responses in a variety of histologies and a favorable safety profile [27].

The majority of trials look at Dostarlimab in combination with PARP inhibitors, antiangiogenic medicines, chemotherapy, or other immunotherapies such as TSR-022 (anti-TIM-3) or TSR-033 (anti-LAG-3). There have been no efficacy results released yet; however, a preliminary safety profile report from the AMBER phase I trial (NCT02817633, Dostarlimab plus TSR-022, an anti-TIM-3) has been released.

Studies show that the doublet and triplet combination of Dostarlimab with Niraparib or Carboplatin-Paclitaxel, with or without Bevacizumab, was safe and tolerable with promising evidence of antitumor activity in patients with advanced solid tumors. The co-administration of Niraparib, Carboplatin-Paclitaxel, or Bevacizumab did not affect the PK of Dostarlimab. To prevent the potential impact of prior medications on the efficacy of the combinations, we tested them in PARP inhibitor-naive and PD-1/L1 inhibitor-naive patients [14]. Combining these therapies with PD-1/PD-L1 increases several processes in the cancer-immunity cycle, reshapes the TME, and accelerates the transition from non-inflamed to inflamed tumors significantly.

### 2.3. Detection of High-Grade Microsatellite Instability (MSI-H) or Underlying Deficient Mismatch Repair Protein (dMMR)

MSI-H/dMMR is found in 13–30% of recurrent endometrial malignancies. The mutations that cause dMMR endometrial malignancies are mostly somatic (90%) in nature, with 5–10% of cases involving germline alterations [28]. Cancers with dMMR mutations can upregulate the expression of programmed death receptor-1 (PD-1) ligands 1 and 2 (PD-L1 and -L2), which are present on T-cells and limit proliferation and cytokine production when activated. These ligands bind to PD-1, which acts as an immunological checkpoint that suppresses the anti-tumor immune response. Dostarlimab is a monoclonal antibody that binds to the PD-1 receptor and blocks it from interacting with PD-L1 and PD-L2, allowing the anti-tumor immune response to continue unhindered.

The mismatch repair mechanism is a crucial step in the preservation of genomic integrity. It is involved in processes including mitotic and meiotic recombination, immunoglobulin gene rearrangement, apoptosis, and more. Testing for d-MMR or MSI-H helps to identify patients who are likely to respond to PD-1 inhibitors [28,29]. Endometrial carcinoma, colon adenocarcinoma, and stomach adenocarcinoma have the greatest frequencies of d-MMR.

## 3. Endometrial Cancer 

Endometrial cancer is the 6th most occurring cancer in women. In 2021, there were more than 400,000 new cases. Patients with advanced and recurrent disease have a dismal prognosis with an expected 5-year survival of less than 20% and limited treatment options. Patients with metastatic disease are eligible for platinum-based chemotherapy. The expected median progression-free survival (PFS) is of 13 months [30]. EC has a tumor type associated with high rates of MSI-H/dMMR. MSI-H/dMMR tumors have a 100–1000-fold increase in mutation rates and express high quantities of neoantigens due to their inability to repair DNA replication mistakes, rendering them immunogenic. Patients with MSI-H/dMMR tumors may be predisposed to respond to PD-1 and PD-L1 drugs.

Dostarlimab binds with high affinity to the PD-1 receptor and effectively blocks the interaction with PD-L1 and PD-L2 [31]. The GARNET trial (NCT02715284) sought to determine the safety, tolerability, and anticancer efficacy of Dostarlimab monotherapy in patients with advanced solid malignancies. This study is based on patients who were identified as having dMMR tumors by local immunohistochemically testing. Patients must have shown disease progression during or after platinum-based doublet chemotherapy, and no more than two lines of therapy for the advanced or recurrent disease should have been used.

Most treatment-related adverse events (TRAEs) in the 104 patients included in the safety analysis were grade 1 or 2. Asthenia, diarrhea, tiredness, and nausea were the most reported TRAEs of any grade (10%). TRAEs of grade 3 or above were recorded in 11.5% of cases (*n* = 12), with anemia being the most frequently reported TRAE at 2.9% (*n* = 3). At least one significant TRAE was experienced by 10 patients (9.6%). Colitis was the most frequently reported serious TRAE (2 (1.9%)). Two individuals (1.9%) dropped out of the research due to a TRAE (increased transaminase levels); one of these two patients also exhibited elevated glutamyl transferase levels. There have been no TRAE-related deaths documented. Pneumonitis was detected in one patient, but no grade 3 or higher infections were found.

The dosing regimen used in this clinical trial is a unique feature of Dostarlimab therapy, in addition to its activity. The dose schedule was chosen based on the individual and pharmacokinetic studies, indicating that it provides adequate serum concentrations. After 12 weeks of initial Dostarlimab treatment, it was possible to observe that this innovative dose schedule allows for fewer clinic visits, which helps both patients and caregivers and has the potential to save healthcare expenditures. 

Patients in this cohort were chosen based on their MMR status, which is presently the most reliable predictor of checkpoint inhibitor activity in EC. Other potential indicators, such as PD-L1 expression level and tumor mutational load, were not tested at this time; therefore, our findings and conclusions are limited [32]. This knowledge may aid in identifying patients who will benefit the most from Dostarlimab and, conversely, may aid in identifying potential mechanisms of Dostarlimab resistance in dMMR malignancies [30]. Moreover, using the MMRd predictive biomarker to select patients with EC for immune checkpoint inhibitors could lead to more efficient and sustainable health systems and the avoidance of more harmful combinations, resulting in individualized therapy.

## 4. Rectal Cancer

The conventional treatment for locally advanced rectal cancer is neoadjuvant chemotherapy and radiation followed by rectum surgical resection. Mismatch repair deficit is responsible for a fraction of rectal cancers. A recent study was conducted based on the prediction that checkpoint blockade could be helpful in individuals with mismatch repair-deficient, locally progressed rectal cancer because mismatch repair-deficient colorectal cancer responds to PD-1 blockade in the context of metastatic disease [33]. The neoadjuvant therapy, Fluoropyrimidine, in conjunction with Oxaliplatin is followed by chemoradiotherapy and finally surgery. This treatment produces a pathological full response in up to a quarter of patients, but it is accompanied by significant problems and toxic effects in a significant number of patients, including bowel, urinary, and sexual dysfunction, among others.

In a recent clinical trial, NCT04165772, the following key eligibility criteria were determinant of Dostarlimab success: no evidence of distant metastases, no previous treatment with immunotherapy, chemotherapy or radiation for the rectal tumor, and no active autoimmune or infectious disease or treatment with immunosuppressive therapy. Patients with a clinical complete response underwent no operative follow-up. The lack of residual disease on digital and endoscopic rectal examinations, as well as the absence of residual illness on rectal MRI, with no limited diffusion on T2-weighted imaging, was regarded as a clinical complete response. The overall response to neoadjuvant Dostarlimab therapy with or without chemoradiotherapy satisfied the criteria for the primary endpoint. In 12 consecutive patients who had completed 6 months of therapy, the percentage of patients who had a clinical complete response was 100% [4]. During the 12-month median follow-up period, no patients received chemoradiotherapy, and no patients underwent surgical resection. The pathophysiological full response was not assessed because none of the 12 patients who completed 6 months of Dostarlimab medication had surgery. Furthermore, no illness progression or recurrence occurred in any of the 16 patients that were enrolled, and they are all still alive. The major endpoint for response durability (sustained clinical complete response at 12 months) is not included in the finality of the study. The therapeutic response was quick, with 81% of patients experiencing symptom relief within 9 weeks of starting Dostarlimab. Five patients had an endoscopic complete response at the 3-month examination, but only two had a radiographic complete response. In 12 of the 16 patients, there were adverse events of any severity (75%, 95% CI, 48–92). There were no adversity incidents of grade 3 or higher reported. In one instance, aberrant thyroid function was discovered but that represented 6% of the adversities.

Neoadjuvant immunotherapy has been studied in a variety of solid tumors, including those that are known to be sensitive to checkpoint blockade in the context of metastatic illness, such as NSCLC, urothelial carcinoma, and melanoma. The levels of activity reported in those tumor types were nowhere near as high as the levels seen in people with mismatch repair-deficient rectal cancer. One possible contributing aspect is that we administered 6 months of immunotherapy, whereas the other research looked at shorter checkpoint blockade exposures. In mismatch repair-deficient tumors, immunotherapy responses have been found to evolve over months rather than weeks. Why these localized mismatch repair-deficient rectal tumors respond so much better than metastatic colorectal malignancies is an interesting topic. Despite the presence of molecular features at baseline that was like those of the tumors evaluated in our study, the rate of imaging-based complete response of mismatch repair-deficient colorectal tumors was 11.1% in a study involving patients with metastatic disease who had not previously received any treatment.

The potential influence of the gut microbiome on cancers of the gastrointestinal system was hypothesized. An increasing body of evidence supports the immunomodulatory role of specific bacterial species in enhancing the anti-tumor immune response, which is boosted by checkpoint blockade. Although the results of our study are promising, especially considering that 12 consecutive patients had a clinical full response, the major study limitations are as follows: the study is small and only represents the experience of one institution. These findings need to be replicated in a larger prospective cohort that includes patients from a variety of racial and ethnic backgrounds and balances academic and community practices [4].

In the first line setting for patients with dMMR/MSI-H disease, Pembrolizumab has been approved as the preferred option, and Nivolumab, alone or in combination with Ipilimumab, has been approved as an alternative option for patients with dMMR/MSI-H disease, regardless of their eligibility for intensive chemotherapy. Both these immunotherapeutic regimens (e.g., Pembrolizumab and Nivolumab +/− Ipilimumab) and Dostarlimab are now indicated for patients with dMMR/MSI-H chemoresistant metastatic colorectal cancer (in patients who have not previously received an ICI). Focusing on the premise of targeting immune-mediated interaction in the dMMR/MSI-H intestinal milieu.

Targeting molecular abnormalities seen across diverse tumor histology is becoming increasingly important in cancer treatment. While some oncogenic drivers, such as microsatellite instability (MSI) and NTRK fusions, can be treated the same way regardless of tumor type (“histology-agnostic”), others require histology-specific therapeutic adjustments (“histology-tuned”), which can be accomplished by using specific inhibitors and ad hoc combinations.

Pembrolizumab or Dostarlimab, among histology-agnostic medicines, showed equivalent action in MSI metastatic colorectal cancer (mCRC) as in other MSI tumors, while Entrectinib or Larotrectinib were successful in NTRK rearranged mCRC, albeit less significantly than in the general population. BRAFV600E mutations and ERBB2 amplification are targeted by histology-tuned methods in mCRC, underscoring the need for simultaneous anti-EGFR inhibition or cautious selection of companion medicines in this tumor type. Anti-RET and anti-ALK medicines have emerged as possible histology-agnostic treatments, whereas anti-KRASG12C methods could become histology-tuned therapies in the future. The effects of targeting ERBB2 mutations and NRG1 fusions were mixed. To summarize, agnostic targets such as MSI and NTRK fusions have previously been exploited in mCRC, whereas the multitude of developing histology-tuned targets represent a challenging potential that will necessitate the evolution of molecular diagnostic tools at the same time [34].

## 5. Conclusions 

In MMR-deficient instances, Dostarlimab has demonstrated promising benefits, and the combination of Pembrolizumab and Lenvatinib is quickly becoming the standard of therapy for pre-treated recurrent MMR-proficient EC. However, further research is needed to understand the primary and secondary mechanisms of immunotherapy resistance, as well as to use ICI in the first-line metastatic context and early-stage malignancies. The same applies to rectal cancer. Future clinical trials should go through safety studies to identify higher-risk categories.

Treatments such as Dostarlimab should become widely available, as well as access to a medical team who will help monitor patients like in the trial NCT04165772 and intervene if the tumor comes back. We believe that the future of cancer treatment is an approach based on cancer type and subtype, and such a dramatic response as seen with Dostarlimab in patients with rectal cancer gives hope that we are on the right track to find a dramatic match for the remaining cancers.

## Figures and Tables

**Table 1:** FDA-approved immune checkpoint blocking antibodies.

Target	Antibody Drug	Trade Name	Tumor Type (FDA Approval Year)	Genetic Testing
PD-1	Nivolumab (IgG4)	Opdivo	Melanoma (2014)	BRAF V600
			Nonsmall cell lung cancer (2015)	EGFR or ALK gene
			Hodgkin lymphoma (2016)	
			Head and neck squamous cell carcinoma (2016)	
			Urothelial carcinoma (2017)	
			Hepatocellular carcinoma (2017)	
			Colorectal cancer (2017)	dMMR/MSI-H
			Gastric (stomach) cancer, gastroesophageal junction adenocarcinoma, or esophageal cancer (2021)	
			Renal cell carcinoma (2021)	
	Pembrolizumab (IgG4)	Keytruda	Melanoma (2014)	BRAFV600
			Nonsmall cell lung cancer (2015)	EGFR gene or ALK gene
			Head and neck squamous cell carcinoma (2016)	
			Hodgkin lymphoma (2017)	
			Urothelial carcinoma (2017)	
			Gastric and gastroesophageal carcinoma (2017)	HER2
	Cemiplimab (IgG4)	Libtayo	Cutaneous squamous cell carcinoma (2018)	
			Basal cell carcinoma (2021)	
			Nonsmall cell lung cancer (2021)	EGFR, ALK, or ROS1
	Dostarlimab (IgG4)	Jemperli	Endometrial Cancer and Recurrent or Advanced Solid Tumors (2021)	dMMR/MSI-H
PD-L1	Atezolizumab (IgG1)	Tecentriq	Urothelial carcinoma (2016)	
			Nonsmall cell lung cancer (2016)	EGFR gene or the ALK gene
			Hepatocellular carcinoma (2020)	
			Melanoma (2020)	BRAF
			Small cell cancer (2021)	
	Durvalumab (IgG1)	Imfinzi	Urothelial carcinoma (2017)	
			Nonsmall cell lung cancer (2018)	
	Avelumab (IgG1)	Bavencio	Merkel cell carcinoma (2017)	
			Urothelial carcinoma (2017)	
			Renal cell carcinoma (2019)	
CTLA-4	Ipilimumab (IgG1)	Yervoy	Melanoma (2011)	
			Renal cell carcinoma (2018)	
			Nonsmall cell lung cancer	EGRF or ALK
			Malignant pleural mesothelioma	
			Hepatocellular carcinoma	
			Colorectal cancer	dMMR/MSI-H

**Table 2:** Clinical trials testing the combination of drug Dostarlimab with other therapies.

Target Population	Combination	Clinical Trial
Endometrial cancer	Dostarlimab and niraparib	NCT03016338
Head and neck cancer	Dostarlimab and niraparib	NCT04313504
Localized unresectable adult primary liver cancer	Dostarlimab and TSR-022	NCT03680508
Melanoma stage III or IV	Dostarlimab and TSR-022	NCT04139902
Endometrial or ovarian carcinosarcoma	Dostarlimab and niraparib	NCT03651206
Recurrent ovarian cancer	Dostarlimab and niraparib	NCT03806049
Stage III or IV nonmucinous	Standard of care ± dostarlimab and niraparib	NCT03602859
Advanced (unresectable) or metastatic solid tumor	Dostarlimab and TSR-022 (anti-TIM-3)	NCT02817633
Advanced (unresectable) or metastatic solid tumor	Dostarlimab and anti-LAG-3	NCT03250832
Mainly NSCLC or any other metastatic cancer	Dostarlimab and TSR-022 (combination), platinum-based doublet chemotherapy, bevacizumab and niraparib	NCT03307785
Recurrent ovarian cancer	Dostarlimab, niraparib and bevacizumab	NCT03574779
Advanced and metastatic NSCLC	Niraparib + pembrolizumab/dostarlimab	NCT03308942
Ovarian advanced cancer	Dostarlimab and niraparib	NCT03955471
Triple negative breast cancer	Dostarlimab and Niraparib plus radiation therapy	NCT04837209
Advanced Nonsmall Cell Lung Cancer	Dostarlimab and Cobolimab	NCT04655976
Metastatic Non-Squamous Nonsmall Cell Lung Cancer	Dostarlimab and chemotherapy (pemetrexed, cisplatin, and carboplatin)	NCT04581824
Relapsed/Refractory Multiple Myeloma	Dostarlimab and Belantamab mafodotin	NCT04126200

## Data Availability

Not applicable.

## Abbreviations

(dMMR)	mismatch repair deficient
(FDA)	Food and Drug Administration
(HCL)	hairy cell leukemia
(IL-2)	interleukin-2
(EU)	European Union
(US)	United States
(PD-1)	programmed cell death protein-1
(CTLA-4)	cytotoxic T -lymphocyte-associated protein-4
(ICPIs)	immune checkpoint inhibitors
(PD-L1 and PD-L2)	programmed cell death ligands 1 and 2
(CHO)	Chinese hamster ovary
(ADCC)	antibody-dependent cellular cytotoxicity
(CDC)	complement-dependent cytotoxicity
(ADA)	anti-drug antibodies
(NSCLC)	nonsmall cell lung cancer
(PBMCs)	human peripheral blood mononuclear cells
(PopPk)	population PK
(CL)	clearance
(ER)	exposure-response
(AEs)	adverse events
(TMBST)	tumor mutational burden status
(ORR)	overall response rate
(APCs)	antigen-presenting cells
(NAbs)	neutralizing antibodies
(CRs)	complete responses
(iE)	Induced encephalitis
(BCMA)	B-cell maturation antigen
(PFS)	progression-free survival
(TRAEs)	treatment-related adverse events
(mCRC)	colorectal cancer
